# An analysis of the COVID-19 laboratory dataset at AiBST Laboratory in Harare, Zimbabwe, 2020

**DOI:** 10.11604/pamj.2021.40.183.28520

**Published:** 2021-11-25

**Authors:** Tendai Chipendo, Takudzwa Marembo, Humphrey Chituri, Clayton Munemo, Portia Manangazira, Donewell Bangure, Justen Manasa

**Affiliations:** 1 Africa Centres for Disease Control and Prevention, African Union Commission, Addis Ababa, Ethiopia,; 2 Department of Medical Microbiology, Faculty of Medicine, Midlands State University, Gweru, Zimbabwe,; 3 Epidemiology and Disease Control Directorate, Ministry of Health and Child Care, Harare, Zimbabwe,; 4 Department of Laboratory Medicine and Investigative Sciences, Faculty of Medicine and Health Sciences, University of Zimbabwe, Harare, Zimbabwe,; 5 African Institute of Biomedical Science and Technology, Harare, Zimbabwe

**Keywords:** Coronavirus disease 2019 (COVID-19), pandemic, laboratory, Zimbabwe

## Abstract

**Introduction:**

coronavirus disease 2019 (COVID-19) has become a major public health problem and has spread rapidly around the globe since its first identification in Wuhan, China, in December 2019. Zimbabwe reported its first case on the 20^th^ March 2020, and since then the disease has spread to almost every part of the country. Laboratory testing is important in controlling this pandemic. However, few studies have focused on assessing trends of SARS-CoV-2 laboratory data. We described SARS-CoV-2 data from African Institute of Biomedical Science and Technology (AiBST) Laboratory in Harare, Zimbabwe.

**Methods:**

a retrospective record review of secondary SARS-CoV-2 data from AiBST Laboratory in Harare between May to September 2020 was done. Epi Info TM 7.2.2.6 was used to generate frequencies, proportions and conduct bivariate analysis.

**Results:**

a total of 6,535 SARS-CoV-2 laboratory records were analysed. The median age of the patients was 36 years and 55% (3594/6535) were males. There was an increase in average analytical turn-around time (TAT) of SARS-CoV-2 results from May to August 2020. Analytical and preanalytical TAT remained above 2 days from August to September. Males were 1.18 times at risk of being SARS-CoV-2 infected than females (p<0.05). The risk of being SARS-CoV-2 infected increased with age from 1.06 in the 11-20 age group to 1.45 in the 41-50 age group.

**Conclusion:**

COVID-19 poses a greater threat to the older age groups and to men. The delayed TAT of SARS-CoV-2 results limits the efforts to control the pandemic. Decentralization of testing to provincial and district level would help improve result TAT.

## Introduction

Coronavirus disease 2019 (COVID-19) is a communicable respiratory disease caused by a novel coronavirus named severe acute respiratory syndrome coronavirus 2 (SARS-CoV-2) [1]. The initial cases of COVID-19 were identified in Wuhan City, Hubei Province, China and reported to the World Health Organisation (WHO) on December 31^st^, 2019. Since then, the disease has spread to almost every region and country in the world and was declared a COVID-19 global pandemic by the WHO on March 11^th^, 2020 [2].

Over 100 million cases have been reported globally with approximately 2.3 million deaths as of 31^st^ January, 2021 [3]. Zimbabwe recorded its initial case on the 20^th^ of March 2020 and has recorded 33,388 cases and 1,217 deaths as of 31^st^ January, 2021 [4]. COVID-19 virus spreads from person to person through infected air droplets that are projected during sneezing or coughing. It can also be transmitted when humans have contact with hands or surfaces that contain the virus and touch their eyes, nose or mouth with contaminated hands.

The disease has a wide range of clinical presentations, from mild symptoms resembling the common flu to severe, life-threatening manifestations such as adult respiratory distress syndrome (ARDS), thrombotic complications and neurological symptoms [5]. Risk factors for adverse outcomes include hypertension, diabetes, cardiovascular disease and respiratory disease [5]. In the clinical management and control of the COVID-19 disease, rapid collection and laboratory testing of appropriate specimens is very critical. Early diagnosis permits physicians to provide prompt intervention for patients who are at higher risk for developing more serious complications from COVID-19 illness and minimizes spread of infection by positive individuals [6].

The Ministry of Health and Child Care in Zimbabwe adopted the WHO recommended testing strategy which involves implementation of prioritized testing and measures that can reduce spread such as isolation [7]. Reverse transcription polymerase chain reaction (RT-PCR) is the gold standard for laboratory diagnosis of SARS-CoV-2 infection and a number of platforms are being used for real-time PCR at both public health and private laboratories.

With testing having emerged as a major tool in the effort to battle the ongoing COVID-19 pandemic, more effective use of clinical laboratory data is important in managing the disease. SARS-CoV-2 has presented with challenges where scientists and clinicians are struggling to understand not just the mechanisms and characteristics of the virus but also its movement and prevalence within affected populations and where laboratory testing is one of the main sources of insight into the virus´s impact and behaviour. An analysis of the laboratory dataset helps to guide the response to the virus, enabling the tracking of the progression of the pandemic and deployment of resources more efficiently. There is no documented evidence that the laboratory data has been analysed before. We therefore analysed the COVID-19 data collected from May to September 2020 at AiBST Laboratory in Harare.

## Methods

**Study design:** a retrospective descriptive cross-sectional study based on secondary COVID-19 data from AiBST Laboratory in Harare was done.

**Study setting:** the study was conducted between May and September 2020 using data from AiBST Laboratory in Harare. This is the period when Zimbabwe experienced its first wave of SARS-CoV-2. The laboratory is one of the major research and education institutes in Zimbabwe carrying out a wide range of activities ranging from molecular diagnostics to forensic science. The laboratory´s main catchment area is Harare, with a population of approximately two million people basing on the 2012 census projections [8]. There is an adequate staff complement of proficient scientists with great capacity for quality testing and an electronic information system to store and manage data. The laboratory receives COVID-19 specimens for RT-PCR from both private and public health institutions across Zimbabwe.

**Data source:** the COVID-19 data from the laboratory request forms were captured and stored in the laboratory information management system (LIMS). The dataset included information on COVID-19 testing results, categorised as positive and negative, date of specimen collection, date of result dispatch as well as individual level data on demographic characteristics and facility specific information. Demographic information included patients´ age and gender. No data on the clinical characteristics was available. The dataset was created in May 2020 and records from May to September 2020 were analyzed.

**Study variables:** as per the country COVID-19 laboratory policy, the laboratory was expected to perform RT-PCR assays for the detection of SARS-CoV-2. The outcome variable of interest in the study was whether a patient was diagnosed with SARS-CoV-2 infection or not, defined as a dichotomous indicator. We thus excluded 86 patients (1.3%) with pending and invalid results, resulting in a final sample of 6,535 patients. We included individual level, demographic characteristics (age and gender) and facility level information including the province from which the sample had been referred. Dates of specimen collection, receipt at the laboratory and results dispatch were used to determine the preanalytical and analytical turnaround time (TAT). Pre-analytical TAT was defined as number of days from the date of specimen collection to date the sample is received at the laboratory. Analytical TAT was defined as the number of days from date of specimen receipt at the laboratory to date of result release.

**Data analysis:** data quality was checked using completeness of data entries in LIMS. Epi Info ™ 7.2.2.6 software was used to generate frequencies, measures of central tendency and proportions of the different characteristics of the COVID-19 records. To estimate the association of the demographic characteristics with the outcome of interest (SARS-CoV-2 positive diagnosis for all patients), we conducted bivariate analysis using the same software.

**Ethical considerations:** the names of SARS-CoV-2 patients were not captured. An anonymous identification number was assigned to each record during data analysis. Adequate administrative authorization was obtained to conduct the study.

## Results

A total of 6,535 COVID-19 laboratory records of samples processed between May and September 2020 were analyzed. The overall completeness of the dataset was 98.7%.

**Demographic characteristics:** the median age of the patients was 36 years and the majority (31%) were in the 31-40 years age group. Fifty five percent (3594) of the patients were males while 45% were females. Sixty one percent (4016) of the patients were from Harare Province, 14% (925) from Mashonaland East while only 3% (193) were from Manicaland Province (Table 1).

**Table 1:** demographic characteristics of patients tested for COVID-19 at AiBST Laboratory in Harare, May to September 2020 (n=6535)

Variable	Category	Frequency n (%)
Sex	Males	3594 (55)
	Females	2941 (45)
Age group (years)	≤ 10	290 (4)
	11-20	380 (6)
	21-30	1437 (22)
	31-40	2011 (31)
	41-50	1311 (20)
	51+	1106 (17)
Median age (years)	36 (Q^1^ = 28; Q^3^ =45)	
Province	Harare	4016 (61)
	Mashonaland east	925 (14)
	Mashonaland west	476 (7)
	Mashonaland central	368 (6)
	Masvingo	291 (4)
	Midlands	266 (4)
	Manicaland	193 (3)

**Figure 1:**
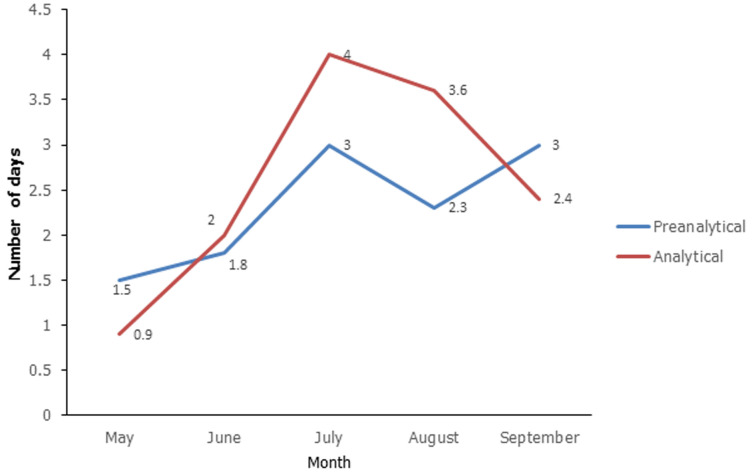
average pre-analytical and analytical turn around time of SARS-CoV-2 results, Harare, May to September 2020 (source: laboratory information management system)

**Turn around time of COVID-19 results:** the average preanalytical and analytical turn-around time of SARS-CoV-2 results from May to September is shown in Figure 1. There was an increase in the average preanalytical turn-around time from 1.5 days to 3 days from May to July which decreased to approximately 2 days in August. The average analytical TAT increased from 1 day to 4 days from May to August and decreased to 2 days in September.

**Trends in positivity:** out of the 6,535 samples tested for SARS-CoV-2 at the laboratory, 805 (12.3%) tested positive while 5,730 (87.7%) were negative. Harare Province had the highest proportion (55%) of SARS-CoV-2 infected cases, followed by Mashonaland East Province (19%). Manicaland Province had the lowest proportion of cases (1%).

**Figure 2:**
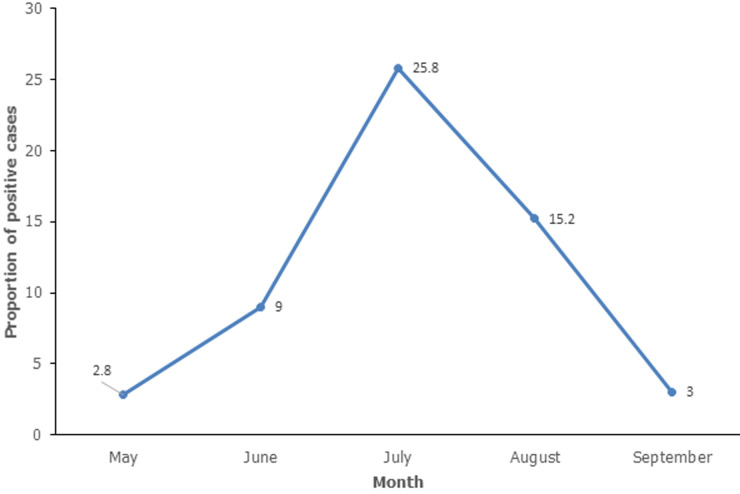
distribution of SARS-CoV-2 cases by month, Harare, May to September 2020 (n=805) (source: laboratory information management system)

**Distribution of SARS-CoV-2 infected cases by month:** there was an increase in the proportion of SARS-CoV-2 infected cases from 2.8% to 25.8% from May to August. The positivity rate decreased to 9.7% in September (Figure 2).

**Distribution of positive cases by sex and age group:** fifty-nine percent (475) of the cases were males, while 41% (330) were females. The majority (32%) of the cases were in 31-40 years age group. The 0-10 and the 11-20 age groups had the least proportion of cases, 5% and 6% respectively.

**Demographic factors associated with being a SARS-CoV-2 infected case:** males were 1.18 times at risk of being SARS-CoV-2 infected cases than females and this was statistically significant (p<0.05). The risk of being SARS-CoV-2 infected increased with age from the 11-20 age group (OR: 1.06, 95% CI: 0.68-1.67) to the 41-50 age group (OR: 1.45, 95% CI: 1.01-2.10). Those in the 41-50 age group were 1.45 times at risk of being a COVID-19 case with reference to the 0-10 age group and this was statistically significant (p<0.05) (Table 2).

**Table 2:** demographic factors associated with SARS-CoV-2 infections, Harare, 2020

Variable	Category	Positive n=805 (%)	Negative n=5730 (%)	OR	95% CI	p value
Sex	Male	473 (59)	3152 (55)	1.18	1.02-1.38	0.01*
	Female	332 (41)	2578 (45)			
Age group	0-10 (ref)	43 (5)	254 (4)	1		
	11-20	48 (6)	307 (5)	1.06	0.68-1.67	0.38
	21-30	188 (23)	1249 (22)	1.10	0.77-1.58	0.28
	31-40	257 (32)	1754 (31)	1.14	0.81-1.60	0.23
	41-50	135 (17)	1176 (21)	1.45	1.01-2.10	0.02*
	51+	134 (17)	806 (17)	1.23	0.85-1.78	0.13

## Discussion

In our study, we noted a prolonged TAT of SARS-CoV-2 results against the 48 hours set by the National Microbiology Reference Laboratory in the period under review. The average preanalytical TAT was above 1 day from May to September and was as high as 3 days in July and September. A longer preanalytical TAT means a delay in the samples reaching the laboratory for testing hence a delay in testing and release of results. A number of factors could have contributed to the long preanalytical TAT including, transport and fuel challenges from the referring health facilities to the SARS-CoV-2 testing laboratory. During the period of the first SARS-CoV-2 wave in Zimbabwe, the effected lockdown affected the smooth flow of a number of service providers.

The increase in analytical TAT observed from May to August could be due to the increase in the sample flow to the laboratory, which coincided with an expansion of the testing strategy as the country entered into the phase of community SARS-CoV-2 transmission. As more samples were being received at the laboratory, workload and burden to the laboratory increased resulting in proportional increase in analytical TAT.

Furthermore, the increase in the analytical TAT could also have been due to the scarcity and erratic supply of some laboratory commodities needed for testing. The automated test method suffered from a lack of test kits, because of insufficient production capacity to meet the demand and global competition. Laboratories were forced to use non-automated test methods, which are labour-intensive, require skilled and trained staff and have low- to-medium throughput. Similar findings were noted in a study by Porter *et al*. (2020) in South Africa where the use of non-automated test methods was one of the reasons contributing to a longer analytical TAT of SARS-CoV-2 results [9].

A rapid TAT is critical in the control of the COVID-19 pandemic. The spread of the pandemic is limited by finding and isolating SARS-CoV-2 positive cases and quarantining their contacts. However, the prolonged TATs noted in this study could mean that the majority of positive patients were contacted once they were no longer infectious and the majority of their contacts were traced and contacted after they would already have been contagious had they also been infected [9,10].

In this study, we noted an increased risk of being SARS-CoV-2 infection with age. Similar trends have been observed in a number of epidemiological studies, where the elderly have been noted as being more vulnerable to COVID-19 [11-13]. Older people face significant risk of developing severe illness if they contract the virus due to physiological changes that come with ageing and potential underlying health conditions [14]. Contrary to our findings, Gupta *et al*. (2020) noted that the SARS-CoV-2 infections in India were biased towards younger adults more than the hypothesis of uniform attack rate and susceptibility across the whole population would predict [15].

Furthermore, males were found to be 1.18 times at risk of being SARS-CoV-2 infected as compared to females in this study. Several studies have shown a significant association between poorer outcome and being male [16,17]. Although the reasons underlying this observation are not fully understood, the ACE2 level in males has been suggested as a possible explanation [17].

**Limitations:** this study used available data and therefore did not require any extra data collection. Unfortunately, there were several limitations to the study. There were several missing essential data elements, especially the disaggregation by co-morbidities and travel history which would have allowed us to reconstruct a better picture of the trends. Without primary data collection, this weakness is expected.

## Conclusion

The delayed TAT of SARS-CoV-2 results limits the efforts to control the pandemic through delayed contact tracing and isolation of those who test positive. Decentralization of testing to provincial and district level through provision of GeneXpert cartridges and SARS-CoV-2 antigen tests kits would help improve the turnaround time of the results. COVID-19 poses a greater threat to the older age groups and to men. It is necessary to carry out key monitoring, prevention and control of the elderly as well as men, and strengthen early warning and intervention of severe cases.

### What is known about this topic

- Rapid collection and laboratory testing of appropriate specimens is very critical in the clinical management and control of SARS-CoV-2 infections;
- More effective use of clinical laboratory data is important in managing the COVID-19 disease.

### What this study adds

- Monitoring the turnaround time of COVID-19 results is important in controlling the pandemic;
- Efforts to decentralize testing should be made to improve results turnaround time.
